# DWNN, a novel ubiquitin-like domain, implicates RBBP6 in mRNA processing and ubiquitin-like pathways

**DOI:** 10.1186/1472-6807-6-1

**Published:** 2006-01-05

**Authors:** David JR Pugh, Eiso AB, Andrew Faro, Portia T Lutya, Eberhard Hoffmann, D Jasper G Rees

**Affiliations:** 1 Biotechnology Department, University of the Western Cape, Modderdam Road, Bellville 7535, South Africa; 2 Department of NMR Spectroscopy, Bijvoet Center for Biomolecular Research, University of Utrecht, Padualaan 8, 3584 CH Utrecht, The Netherlands; 3 Department of Molecular and Cell Biology, University of the Witwatersrand, 1 Jan Smuts Avenue, Johannesburg 2050, South Africa; 4 Varian Deutschland GmbH, Alsfelder Straβe 3, D-64289 Darmstadt, Germany

## Abstract

**Background:**

RBBP6 is a 250 kDa splicing-associated protein that has been identified as an E3 ligase due to the presence of a RING finger domain. In humans and mice it interacts with both p53 and Rb, and plays a role in the induction of apoptosis and regulation of the cell cycle. RBBP6 has recently been shown to be highly up-regulated in oesophageal cancer, and to be a promising target for immunotherapy against the disease.

**Results:**

We show here using heteronuclear NMR that the N-terminal 81 amino acids of RBBP6 constitute a novel ubiquitin-like domain, which we have called the DWNN domain. The domain lacks conserved equivalents of K^48 ^and K^63^, although the equivalents of K^6 ^and K^29 ^are highly, although not absolutely, conserved. The di-glycine motif that is characteristic of proteins involved in ubiquitination is found in the human and mouse form of the domain, although it is not present in all organisms. It forms part of a three-domain form of RBBP6 containing the DWNN domain, a zinc knuckle and a RING finger domain, which is found in all eukaryotic genomes so far examined, in the majority of cases at single copy number. The domain is also independently expressed in vertebrates as a single domain protein.

**Conclusion:**

DWNN is a novel ubiquitin-like domain found only at the N-terminus of the RBBP6 family of splicing-associated proteins. The ubiquitin-like structure of the domain greatly increases the likelihood that RBBP6 functions through some form of ubiquitin-like modification. Furthermore, the fact that the DWNN domain is independently expressed in higher vertebrates leads us to propose that the domain may itself function as a novel ubiquitin-like modifier of other proteins.

## Background

A number of screens have been undertaken to identify proteins that interact with the tumour suppressor proteins p53 and Rb. RBBP6 is one of the few proteins identified that has been shown to interact with both p53 and Rb. Three partial cDNA's from the full length RBBP6 transcript were originally cloned and sequenced in different studies. RBQ-1 [1], corresponding to residues 150–1146 of the human protein, and PACT (p53 associated cellular protein, testis derived) [2], corresponding to residues 207–1792, were cloned on the basis of their ability to bind Rb and p53 in both human and mouse cells. P2P-R [3], corresponding to residues 199–1792, was cloned based on its recognition by two antibodies specific for heterogeneous nuclear ribonucleoproteins (hnRNPs). An alternatively spliced form omitting residues 651–685, corresponding to exon 16 of the full length gene, has also been reported [1]. The HGMW-approved name Retinoblastoma binding protein 6 (RBBP6) will be used for the complete protein in what follows. Analysis of the RBBP6 locus on human 16p12.2 suggests that three major transcripts of 6.1, 6.0 and 1.1 kb occur, by a combination of alternative splicing and alternative poly-adenylation. These transcripts encode proteins of 1792, 1758 and 118 amino acids, which have been designated RBBP6 isoforms 1, 2 and 3 respectively (Genbank:NP_008841, Genbank:NP_061173, Genbank:NP_116015).

RBBP6 has been shown to suppress the binding of p53 to DNA [2] and to block the binding of the adenovirus E1A protein to Rb [1-3], suggesting that the interactions with both tumour suppressor proteins are biologically relevant. RBBP6 strongly localises to chromosomes during mitosis and to nuclear speckles, which are believed to be the main sites of activity for pre-mRNA splicing and processing, during interphase [4,5]. Over-expression has been shown to lead to cell cycle arrest and apoptosis [6-10]. The yeast homologue, Mpe1p, forms part of the Yeast Cleavage and Polyadenylation Factor and is essential for the specific cleavage and polyadenylation of pre-mRNA [11].

Recently it has been reported that RBBP6 is strongly up-regulated in oesophageal cancer cells, and high levels of expression correlate with higher rates of proliferation in cultured oesophageal cancer cell and low survival rates in cancer patients [4]. Cytotoxic T cells specific for RBBP6-derived peptides were able to lyse oesophageal cancer cells in culture, and to produce regression of oesophageal tumours in mice xenograft models.

Despite its potential as an anti-cancer target and its apparently close association with transcription and the cell cycle, very little is known about the function of RBBP6. In this report we describe the structure of the N-terminal domain of the human RBBP6 protein (which we have named the DWNN domain) and show that it adopts a ubiquitin-like fold. Taken together with the presence of the RING finger domain in all RBBP6 homologues, this result suggests that RBBP6 may regulate pre-mRNA processing proteins by covalently modifying them with a ubiquitin-like moiety. Given that the DWNN domain is expressed as a single domain protein in the vertebrates as the isoform 3 form, the additional possibility exists that the DWNN domain itself plays the role of the ubiquitin-like modifier.

## Results

### The RBBP6 protein family

An investigation using promoter-trapping technology led to the identification of a novel hamster gene homologous to the human cDNA 21c4 (Genbank:T25012) [12]. This was completely sequenced and shown to be a 0.9 kb cDNA clone encoding a 118 amino acid protein (Rees *et al*, unpublished). The corresponding gene was found to be located on human chromosome 16p12.2, upstream of the previously identified RBBP6/PACT/P2P-R gene. Analysis of cDNA sequences showed that the sequence coded for the previously unidentified N-terminus of the RBBP6 protein (Dlamini *et al*, in prep), which we have named the DWNN domain. A number of complete RBBP6 cDNA clones have now been sequenced (Genbank:AB112074, Genbank:AB112075, Genbank:BC029352) and these confirm the presence of three mRNA transcripts of 6.1, 6.0 and 1.1 kb, which occur as the result of a combination of alternative splicing and alternative poly-adenylation. The three transcripts encode proteins of 1792, 1758 and 118 amino acids, which have been designated RBBP6 isoforms 1, 2 and 3 respectively. (Genbank:NP_008841, Genbank:NP_061173, Genbank:NP_116015)

**Figure 1:**
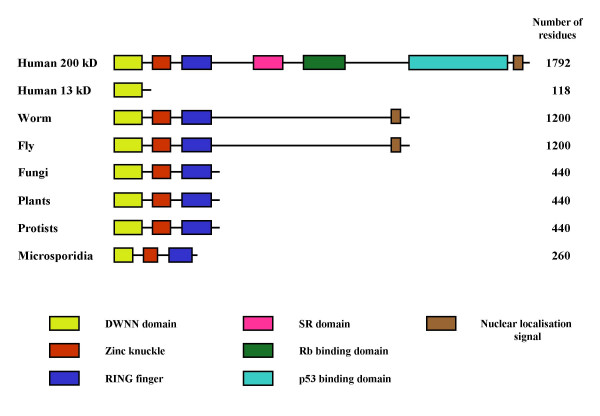
**The domain structure of the RBBP6 family of proteins**. RBBP6 homologues containing a DWNN domain, a zinc knuckle and a RING finger are found in all complete eukaryotic genomes analysed to date, including the single celled parasite *E. cuniculi*, in which it is very much reduced in size. In vertebrates and insects the protein includes a long C-terminal extension containing p53 and Rb-interaction domains in human and mouse. A short form consisting of the DWNN domain and a poorly conserved C-terminal tail is also found in vertebrates.

Extensive BLAST searches [13] against all available sequence data showed that the DWNN domain is found only at the N-terminus of the RBBP6 family of proteins. All of the identified RBBP6 homologues include the DWNN domain, a CCHC zinc finger and a RING finger domain and are found as single copy genes in all complete eukaryotic genomes analysed to date (see Figure 1). The three domain form is found in plants, protozoa, fungi and microsporidia, although it is much reduced in size in the single-celled parasite *Encephalitozoon cuniculi*, which has a highly compacted genome and has been identified as being close to the "minimal" eukaryotic organism [14]. The RBBP6 homologues in vertebrates and insects are longer and include additional domains, including the Rb-binding and p53-binding domains identified previously [1-3]. However, there are no homologous sequences present in prokaryotes.

The zinc finger is of the CCHC type, also known as a "zinc knuckle" [15], which occurs in a number of mRNA-associated proteins, including the splicing factors SLU7, h9G8 and hSF1 [16]. The zinc knuckle from SLU7 has been shown to play an active role in preventing SLU7 from shuttling between the nucleus and the cytosol during mRNA processing [17]. RING fingers are typically found in E3-ubiquitin ligases and have been shown to play an essential role in the conjugation of ubiquitin and ubiquitin-like moieties to protein substrates [18]. The RBBP6 RING domains have a C-X_2_-C-X_11_-C-C-X- [NS]-X_2_-C-X_2_-C-X_12_-C-X_2_-C rather than the classical C3HC4 consensus, which means they are either C4C4 or C3NC4-type RING fingers, depending on which residues are involved in coordinating the two zinc ions. C4C4 RING-like domains have been found in the transcription-associated proteins CNOT4 [19,20] and p44 [21], and despite its non-typical consensus CNOT4 has also been shown to have ubiquitin-ligase activity [20]. In addition to the conserved cysteines, RBBP6 RING domains share the wider set of conserved hydrophobic residues characteristic of U-box domains [22]. These are even stronger predictors of ubiquitin-conjugating function than the metal-chelating residues, since they are shared by a wider set of domains that adopt the same fold and participate in ubiquitination even in the absence of zinc ions.

In addition to the first three domains, human and mouse RBBP6 both contain long C-terminal extensions containing a proline-rich domain (residues 337–349), an SR domain (residues 679–773) and the Rb-binding (residues 964–1120) and p53-binding domains (residues 1142–1727) reported previously [2,3]. Large C-terminal extensions also occur in the *D. melanogaster *and *C. elegans *homologues ([23], Genbank:AF132177, Genbank:NP_492424) although we have not been able to identify clear homologues of the SR, Rb binding and p53 binding domains in these proteins, nor is it known whether they associate with homologues of p53 or Rb.

**Figure 2:**
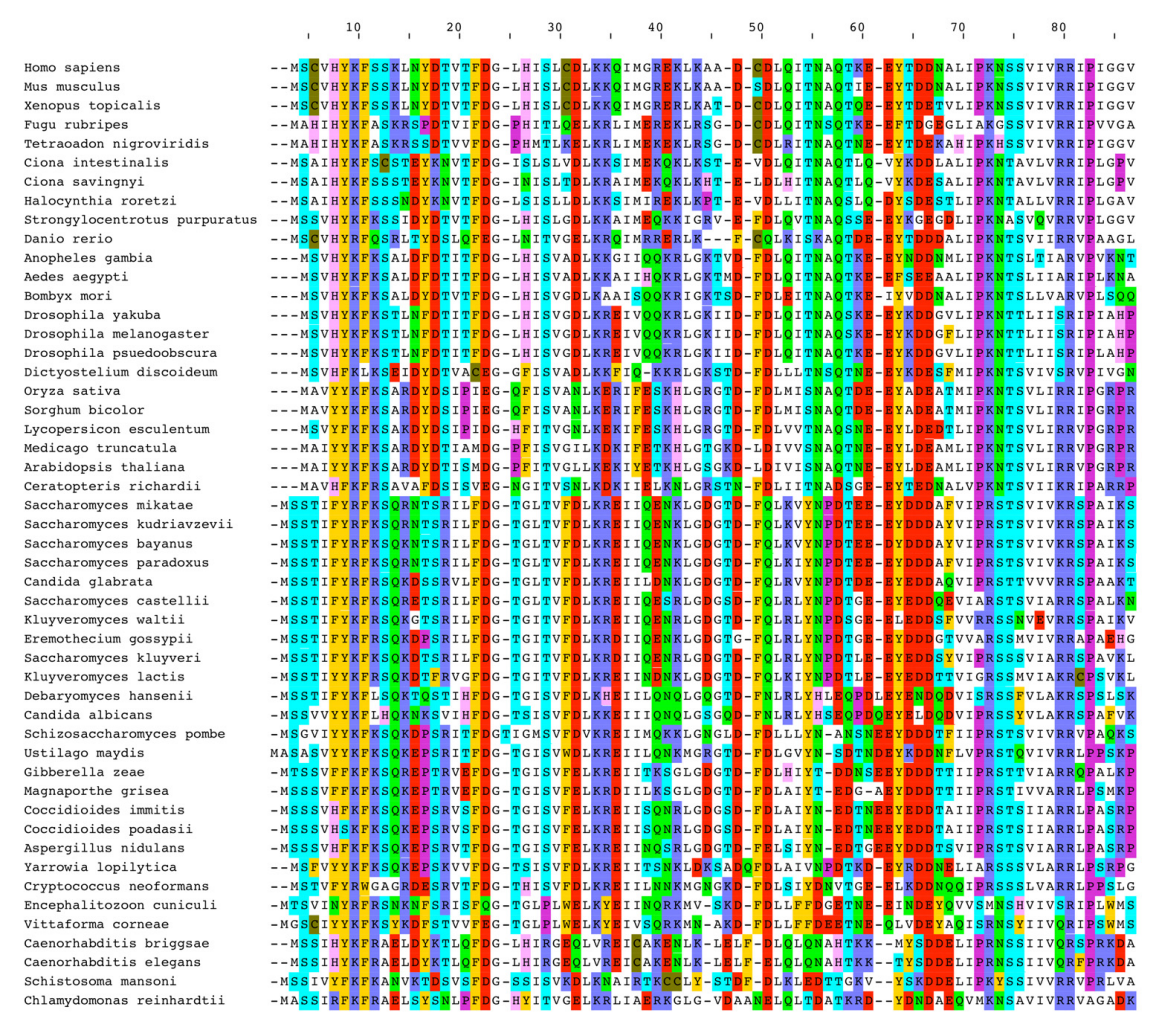
**Sequence alignment of DWNN homologues from a range of eukaryotic genomes**. The DWNN domain has been found in all eukaryotic genomes analysed to date, in the majority of cases at single copy number, but not in prokaryotes. The alignment was performed using ClustalX [39] and the diagram produced using Pfaat [49].

An alignment of DWNN homologues from a range of different eukaryotes is shown in Figure 2. These sequences show no similarity to any other sequences in the database, indicating that the domain represents a novel protein motif that emerged soon after the emergence of eukaryotes. This conclusion is consistent with a comparative genomic study of proteins involved in RNA metabolism [24] showing that the appearance of splicing factors and RNA binding domains such as zinc knuckles coincides with the development of pre-mRNA splicing soon after the emergence of eukaryotes. This is in contrast to proteins involved in basic transcription and translation, many of which share common ancestors which pre-date the divergence of eukaryotes and prokaryotes. In the light of the low copy number at which the RBBP6 gene appears, it is perhaps interesting that many proteins involved in RNA metabolism are also present at low copy number in the genomes of many organisms, despite the high level of conservation and the essential function they fulfil in the organism [24].

In addition to forming part of the full-length RBBP6 protein, the DWNN domain is also expressed in vertebrates as a small protein of 118–150 residues (RBBP6 variant 3) containing a DWNN domain and a short C-terminal tail. Preliminary analysis suggests that the short form is present in all vertebrates but not in invertebrates, plants or fungi. The sequence from residues 82–118 in human is poorly conserved across species, suggesting that it may be un-structured *in vivo*; this conclusion is supported by our observation that this part of the protein is sensitive to proteolysis when expressed recombinantly in bacteria, whereas the DWNN domain (residues 1–81) is highly stable.

### Determination of the structure of the DWNN domain

**Figure 3:**
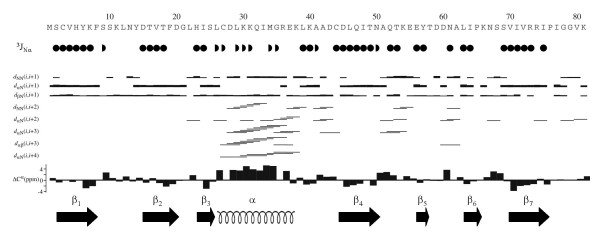
**NOE, chemical shift and coupling constant data used in the structure calculation**. All NOE's were assigned automatically using the CANDID module of CYANA. Cartoon representations of secondary structural elements are derived from the final family of structures. Coupling constants were extracted from a ^15^N-HMQC-J spectrum. Half-circles correspond to ^3^J_Nα _< 4 Hz; filled circles correspond to ^3^J_Nα _> 8 Hz, except in the case of residues 56-7, which have ^3^J_Nα _values of 7.4 and 7.7 Hz respectively. These residues form part of strand β_5_, which is the least stable of the five strands making up the main β-sheet. The presence of a β-sheet comprised of strands β_3 _and β_6_, which does not occur in ubiquitin, is confirmed by large values of ^3^J_Nα_, negative C^α ^shifts and αN(i,i+1) NOE's. The diagram was generated using CYANA [45].

**Figure 4:**
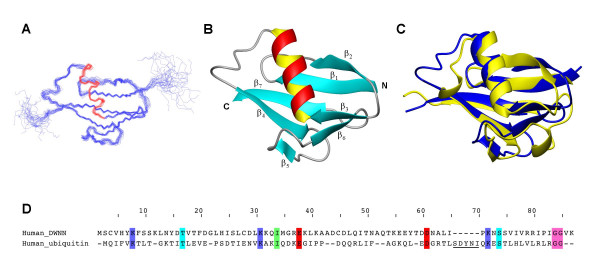
**Three dimensional structure of the DWNN domain**. (A) Superposition of the 25 lowest energy conformers. (B) Cartoon representation of the overall fold and secondary structure. The backbone adopts a ubiquitin-like β-grasp fold in which the central α-helix packs against a five-stranded β-sheet comprised of strands β_1_, β_2_, β_4_, β_5 _and β_7_. Unlike ubiquitin, DWNN contains an additional double-stranded β-sheet at the N-terminal end of the central α-helix, comprising strands β_3 _and β_6_. (C) Superposition of the backbone traces of the DWNN domain (in blue) and ubiquitin (1UBI, in yellow). The RMSD over structurally aligned regions between the two structures is 1.88 Å. (D) Structural alignment of the primary sequences of DWNN and ubiquitin, determined using the Dali server. The structurally equivalent regions comprise the following residues of DWNN: 2–9, 11–20, 22–40, 44–50, 53–57, 59–64, 66–75. Molecular fitting, calculation of RMSD's and generation of figures were performed using MOLMOL [26].

**Figure 5:**
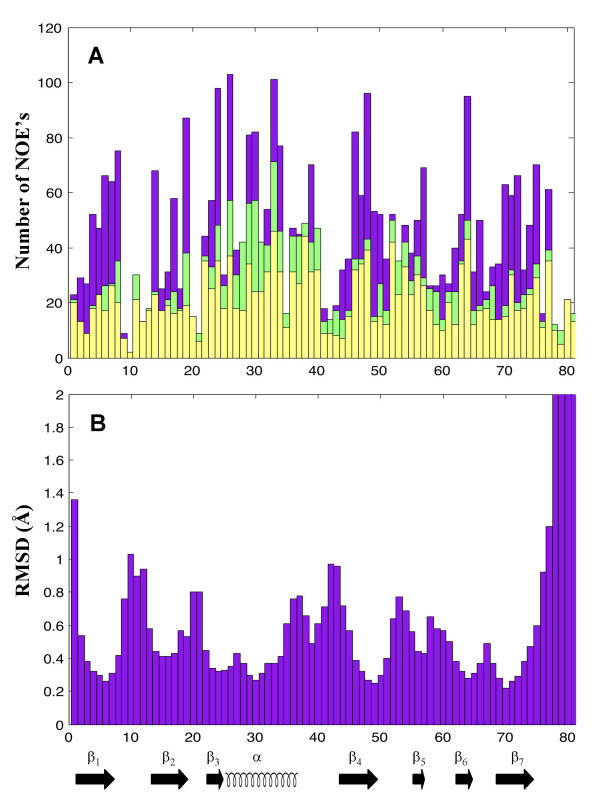
**NOE's and RMSD's broken down by residue**. (A) Distribution of NOE's as a function of residue. Long-range (|i-j|> = 5) – lilac bars; medium-range (1<|i-j|<5) – green bars; short-range (|i-j|< = 1) – yellow bars. NOE's are counted twice – once for each residue involved. (B) RMSD across the family of 25 structures, as a function of residue. Regions of secondary structure correlate well with low RMSD and large numbers of long-range NOE's.

Residues 1–81 of human RBBP6, corresponding to the DWNN domain, were expressed recombinantly in *E. coli *and the structure determined using heteronuclear NMR. Assignment of 99% of all non-labile protons and 90% of ^13^C and ^15^N resonances was achieved using standard triple resonance protocols (for a review see [25]). A total of 1825 NOE-derived distance restraints were used in the structure calculation, made up of 865 short range, 278 medium range and 685 long range restraints. Structural data used in the calculation are summarised in Figure 3. A backbone superposition of the 25 lowest-energy conformers is shown in Figure 4A. The average of the pair-wise root mean square deviations for residues 2–75 is 0.57 Å for all backbone heavy atoms and 1.16 Å for all heavy atoms respectively. Regions of secondary structure were identified in MOLMOL [26] using the built-in algorithm. The number of restraints per residue and the associated local RMSD across the family of structures are shown in Figure 5. As expected, regions of secondary structure correspond to large numbers of long-range NOE's and small RMSD's. The final family of structures have no distance-restraint violations greater than 0.3 Å, no dihedral angle violations greater than 5° and conform well to ideal covalent geometry as determined using the programmes WHATCHECK [27] and PROCHECK-NMR [28]. Statistics relevant to the final family of structures are presented in Table 1.

Comparison of the representative DWNN structure against the entire Protein Data Base using the Dali server [29] reveals that it is most similar to human ubiquitin (PDB:1UBI), and the N-terminal ubiquitin-like domain of Isg15 (PDB:1Z2M), with Z-scores of 7.5 and 7.6 respectively. The amino acid sequences of ubiquitin and DWNN are only 18% identical. A superposition of the backbone traces of the DWNN domain and ubiquitin is shown in Figure 4C, along with a structural alignment of the primary sequences (Figure 4D). The RMSD between the two structures over structurally equivalent regions (see Methods for details of structurally equivalent regions) is 1.88 Å.

The secondary structure consists of the elements β_1_-β_2_-β_3_-α-β_4_- β_5_- β_6_- β_7_, with the α-helix packing against a five-stranded β-sheet made up of strands β_1_, β_2_, β_4_, β_5 _and β_7 _in a ubiquitin-like β-grasp topology (see Figure 4B). Unlike ubiquitin, DWNN contains an additional short section of anti-parallel β-sheet immediately prior to the α-helix (sheets β_3 _and β_6_, residues 23–25 and 63–65 respectively), the evidence for which takes the form of strong α*N*(*i*,*i*+1) NOE's and large ^3^*J*_*N*α _coupling constants (see Figure 3). The residues in this sheet have the highest number of long-range NOE's, and the some of the lowest RMSD values (see Figure 5), suggesting that it is one of the most stable regions of the whole protein. To our knowledge this additional β-sheet has not been seen in other ubiquitin-like proteins. The 3_10 _helix immediately preceding the last β-strand in many ubiquitin-like proteins is not present in DWNN; Figure 4D shows that the residues corresponding to this helix (ubiquitin: 57–61, underlined in Figure 4D) are entirely absent in DWNN. In addition, based on our data we were not able to confirm the presence of a second 3_10 _helix at the C-terminal end of the α-helix that is found in many ubiquitin-like proteins; however the loop preceding strand β_4 _is two residues longer than the corresponding loop in ubiquitin, so that there is no longer a requirement for a tight helical turn at this position. The high level of conservation of G^21 ^(Figure 2) may be the consequence of the presence of the extra strand β_3_, which requires the backbone to make a sharp kink at that position. Hydrophobic residues F^8^, L^29^, I^33^, L^39^, L^46^, I^64^, V^70^, V^72 ^and P^76 ^make up the core of the protein, accounting for their high degree of conservation. The high level of conservation of non-hydrophobic residues Y^6^, K^7^, K^30^, Y^57 ^and R^74 ^suggests a possible functional role for these residues.

## Discussion

In recent years a superfamily of ubiquitin-like domains has been identified [30]. This superfamily can be divided into the ubiquitin-like proteins (UBL's), which consist solely of the ubiquitin-like domain, and ubiquitin domain proteins (UDP's), which are larger proteins containing one or more ubiquitin-like domains. To our knowledge DWNN is the first example of a ubiquitin-like domain that is alternatively expressed both as a UBL and as a UDP.

Ubiquitin-like proteins typically share the C-terminal GG motif, which acts as a recognition motif for a protease that cleaves between the two glycines, initiating the process of conjugation. The occurrence of the GG motif in the structurally identical position in human and mouse DWNN domains (highlighted in pink in Figure 4D) suggests that the domain may be involved in a similar process of conjugation, which we may call "DWNNylation". As in the case of ubiquitin, the GG lies outside of the structured region, as can be clearly seen in Figure 5B. The absence of the GG in lower organisms is more difficult to rationalise; however preliminary EST analysis suggests that organisms which do not contain the GG motif also do not contain the UBL form of the DWNN domain (unpublished data), so it is possible that the DWNN domain does not act as a covalent modifier in lower organisms. In the yeast protein Hub1, which has also been shown to be involved in pre-mRNA splicing [31], the role of the di-glycine motif is taken by a YY motif [32]. The structurally equivalent position in DWNN is taken by a highly conserved RR motif (see Figure 2), which may therefore act as the activation signal.

Ubiquitin contains four conserved lysine residues which are the sites of attachment of additional ubiquitin moieties, leading to the formation of poly-ubiquitin chains in some contexts [33]. K^48^-linked chains are recognised by the 26S proteosome, leading to degradation of the attached protein, whereas K^6^-linked and K^63^-linked chains are involved in a number of non-proteolytic processes, including stress response, DNA repair and endocytosis [34]. K^11^-linked and K^29^-linked chains may also target substrates to the proteosome. In addition, mono-ubiquitination of proteins has been shown to be associated with receptor endocytosis, as well as the sorting and trafficking of proteins [35]. The DWNN domain contains no equivalent of K^48 ^or K^63 ^(see Figure 4D), although the equivalents of K^6 ^and K^29 ^(K^7 ^and K^30 ^in DWNN) are highly, although not absolutely, conserved.

A number of lines of evidence suggest a role for RBBP6 in both mRNA processing and ubiquitin-like protein modification. The close association between domains involved in RNA metabolism and ubiquitination has previously been pointed out in a number of proteins, including MDM2 [24]. In yeast, the RBBP6 homologue Mpe1p has been shown to be a component of the CPF complex [11]. Mammalian RBBP6 has been identified as an SR protein on the basis of an SR domain (residues 477–570) [2], the CCHC RNA binding domain, its localisation within nuclear speckles [9] and its associate with heterogeneous nuclear ribonucleoproteins (hnRNPs) [3]. SR proteins are involved in splicing, whereas hnRNPs are thought to play a central role in organising the polyadenylation, splicing and export of mRNA transcripts [36]. A number of SR proteins are known to interact directly with the C-terminal domain of the RNA Polymerase II complex. A role for RBBP6 in mRNA processing therefore seems highly probable. The presence of a RING finger domain in all eukaryotes, combined with the ubiquitin-like structure of the DWNN domain, makes it highly probable that RBBP6 also has ubiquitin-ligase activity, possibly involving modification of hnRNPs with a ubiquitin-like moiety. Several hnRNPs have recently been shown to be SUMOylated [37], which resulted in a decreased affinity of the hnRNP for mRNA.

Furthermore, since p53 and Rb have both been shown to bind to mammalian RBBP6, it is possible that RBBP6 plays a role in the regulation of these two proteins similar to that played by MDM2 [38], suggesting a possible model for the integration of the regulation of transcription, cell cycle control and apoptosis. Given the fact that the DWNN domain can be independently expressed in vertebrates, an interesting possibility is that the function of RBBP6 is to DWNNylate other proteins.

## Conclusion

We have shown that the DWNN domain represents a novel ubiquitin-like domain expressed in vertebrates both as a single domain protein and as the N-terminus of the RBBP6 family of tumour-suppressor associated proteins, making this the first example of a ubiquitin-like domain that is alternatively expressed both as a UBL and as a UBP.

Members of the RBBP6 family are found at low copy number in all eukaryotes but not in prokaryotes, and the N-terminal three domains (DWNN domain, CCHC zinc knuckle and RING finger domain) are well conserved in all eukaryotes. Longer forms are found in worms, flies and vertebrates, and the human RBBP6 contains domains known to interact with p53 and Rb.

The similarity of DWNN domain to ubiquitin and the presence of the RING finger suggest that the DWNN domain may act as an ubiquitin-like modifier, possibly playing a role in the regulation of the splicing machinery. Whether this involves the proteosome or some other ubiquitin-associated signalling such as regulation of membrane trafficking or protein sorting remains to be determined.

The functional basis for the association with p53 and Rb is less clear. Parallels have been drawn between the vertebrate long form of RBBP6 and MDM2 [2,4] on the basis that both proteins contain RING domains and interact with p53 and Rb. This would suggest a role for RBBP6 in the regulation of the cell cycle and apoptosis and the integration of these processes with transcription and mRNA processing. The presence of the three domain form in even the earliest eukaryotes would appear to support the conclusion that RNA metabolism is the primary role of the protein, and that domains linking the protein to cell cycle regulation were added after the divergence of animals from plants and fungi.

## Methods

### Bioinformatics

Sequences related to the human DWNN domain were identified using BLAST, using all publicly available sequence data [REF BLAST]. 50 examples of DWNN domains from a diverse range of eukaryotes were selected, and sequence alignments were generated using ClustalX [39]. Details of the sequences used in Figure 2 can be found in Additional_data.doc: Table 1.

### Sample preparation

The nucleotide sequence corresponding to residues 1–81 of human RBBP6 was amplified from the 21c4 cDNA [12] using the polymerase chain reaction with forward primer 5'-GAGGCGGGATCCATGTCCTGTGTGCATTATTAATTTTCC-3' and reverse primer 5'-GAGGCGCTCGAGTTATCATTTAACACCTCCAATAGGAAT-3'. The amplified fragment incorporated a *Bam *HI site at the N-terminus, immediately preceding and in-frame with the initiation methionine, and an *Xho *I site at the C-terminus. Two stop-codons (TGA TAA) were inserted between the final codon and the *Xho *I site to prevent read-through. The amplified product was digested with *Bam *HI and *Xho *I and cloned directly into a pGEX-6P-2 vector (GE Healthcare) which had been previously digested with the same enzymes. 1 litre cultures of *E. coli *BL21 (DE3) pLysS cells transformed with the plasmid were grown at 37°C until the OD_550 _reached 0.6, and expression of GST-DWNN fusion protein induced by addition of 0.5 mM IPTG, followed by incubation overnight at 30°C. Cells were pelleted by centrifugation at 3000 × g for 20 mins and re-suspended in 10× mass of Binding Buffer (PBS, 1% TritonX, 1 mM PMSF, 1 mM DTT, pH 7.4). Cells were lysed using the freeze-thaw method (-70°C for 5 min followed by 37°C for 5 min, repeated 3 times), centrifuged at 3000 × g for 30 mins at 4°C and the supernatant loaded onto a self-packed column containing 5 ml glutathione sepharose 4B beads (SIGMA), operated under gravity. GST-DWNN fusion protein was eluted from the column in 5 ml fractions with Elution Buffer (50 mM Tris, pH 8.0, 15 mM reduced glutathione). Fractions containing the fusion protein were dialised back into Cleavage Buffer (50 mM Tris, 150 mM NaCl, 1 mM PMSF, 1 mM DTT, pH 8.0), which had the additional effect of removing free glutathione, and cleaved overnight at 4°C following addition of 1 unit of PreScission™ Protease (GE Healthcare). Following cleavage, GST and PreScission™ Protease were removed by passing the protein a second time down the glutathione sepharose column, with DWNN being collected in the flow-through. Final purification was achieved by concentrating the protein into 1 ml using a Centriprep YM-3 concentration device (Millipore) (MWCO 3000 Da), and loading it onto an 80 cm self-packed Sephacryl S100 gel filtration column (GE Healthcare), which had previously been equilibrated with NMR buffer (100 mM Phosphate, 150 mM NaCl, 1 mM DTT, 0.02% Sodium Azide, pH 6.0). Fractions containing pure DWNN were pooled and concentrated into 0.6 ml for NMR analysis. Despite numerous attempts using a range of different buffers and pH's, we were unable to immobilise the protein on either cation or anion exchange. Protein concentrations were determined using the Bradford Assay [40] employing lysozyme as a standard. Mass spectrometry was used to confirm that the sample was homogeneous and that the protein had the expected molecular weight (data not shown). The expressed protein included five additional residues (GPLGS) at the N-terminus resulting from the cloning procedure; the numbering used in this report corresponds to that in RBBP6.

### NMR experiments

All NMR experiments were carried out at 298 K in aqueous buffer (unless otherwise specified) using triple-resonance probes equipped with z-gradients. A ^13^C-HSQC-NOESY was recorded on a Bruker DRX900; a ^15^N-HMQC-J and a 2D NOESY spectrum in D_2_O were recorded on 500 MHz and 750 MHz Oxford/GE spectrometers respectively; all other spectra were recorded on a Varian Inova600. Spectra were referenced such that the water resonance corresponded to 4.753ppm. NMR spectra were processed using the programme NMRPipe [41] and analysed using NMRView [42].

Sequential assignments were generated using CBCA(CO)NH and CBCANH spectra, in conjunction with a ^15^N-HSQC-TOCSY spectrum to aid in the identification of residues. Side-chain assignments were generated using H(C)CH-TOCSY and H(C)CH-COSY spectra. ^3^J_Nα _couplings were extracted from an HMQC-J spectrum [43] and converted to φ dihedral angle restraints using the standard Karplus relation. Restraints on φ and ψ angles were also generated from N, H^N^, H^α^, C^α^, C^β ^and CO chemical shifts using the TALOS algorithm [44], and these were combined with the coupling constant-derived restraints to give a final set of dihedral restraints. NOE peak volumes were extracted from the following spectra: 2D ^1^H-^1^H NOESY in H_2_O, 2D ^1^H-^1^H NOESY in D_2_O, 3D ^15^N-HSQC-NOESY and 3D ^13^C-HSQC-NOESY.

### Structure calculations

Structure calculations combined with fully-automated NOE assignment were performed using the CANDID module of CYANA v2.0 [45,46]. Each iteration started from a set of 100 random structures, with unassigned NOE peak lists, chemical shift lists and dihedral angle restraints as the only inputs. After each iteration the resulting NOE assignments were statistically analysed and used to generate improved, spectrum-specific chemical shift assignments for use in subsequent iterations. Un-assigned NOE's were analysed manually in combination with NOESY spectra and preliminary structures in order to identify missing chemical shift assignments. The final set of NOE-based restraints, together with dihedral restraints for 90 residues, were used for refinement in explicit solvent using CNS [47], according to the standard RECOORD protocol [48]. Of 100 final structures the 25 lowest energy structures were selected. Validation was carried using WHATCHECK [27] and PROCHECK-NMR [28].

Using the Dali server [29] the following structurally equivalent regions were defined between the DWNN domain and human ubiquitin (PDB:1UBI): DWNN:2–9 ⇔ 1UBI:1–8; DWNN:11–20 ⇔ 1UBI:9–18; DWNN:22–40 ⇔ 1UBI:19–37; DWNN:42–50 ⇔ 1UBI:39–45; DWNN:53–57 ⇔ 1UBI:46–50; DWNN:59–64 ⇔ 1UBI:51–56; DWNN:66–75 ⇔ 1UBI:63–72. The RMSD between the two structures was calculated using all backbone heavy atoms in the structurally equivalent regions. The coordinates of the family of 25 structures have been deposited in the PDB under accession number 2C7H.

## Authors' contributions

DJRP and DJGR conceived of the study. DJRP coordinated the project, participated in the NMR analysis and structure calculations, and drafted the manuscript; EA carried out the structure calculations; AF expressed the protein and participated in the NMR analysis; PTL carried out the molecular biology; EH collected the NMR data; DJGR performed the bioinformatic analysis and helped with the drafting of the manuscript.

## Supplementary Material

Additional File 1 Accession numbers of all sequences used in the multiple alignment in Figure 2. Click here for file

## Figures and Tables

**Table 1:** Statistics of the 25 lowest energy conformations

Restraints	
NOE-derived restraints	
short-range, |i-j|< = 1	865
medium-range, 1<|i-j|<5	297
long-range, |i-j|> = 5	682
	
Dihedral restraints (TALOS)	
φ angle	56
ψ angle	57
	

Final structures	

Pairwise superposition of backbone atoms of residues 2–75	
RMSD over backbone heavy atoms	0.57 Å
RMSD over all heavy atoms	1.16 Å
	
Ramachandran plot appearance	
Most favoured regions	92.32 +- 2.77
Generously allowed regions	6.74 +- 2.47
Generously allowed regions	0.62 +- 0.85
Disallowed regions	0.31 +- 0.57
	
Bad VDW contacts (PROCHECK)	0.00 +- 0.00
	
PROCHECK G-factors	
Dihedrals	-0.21 +- 0.05
Covalent	0.15 +- 0.03
Overall	-0.09 +- 0.04
	
WHATCHECK Z-scores	
1st generation packing quality	-0.34 +- 0.18
2nd generation packing quality	-1.79 +- 0.34
chi-1/chi-2 rotamer normality	-2.10 +- 0.36
Backbone conformation	-1.12 +- 0.47
Bond lengths	0.85 +- 0.02
Omega angle restraints	0.63 +- 0.05
Side chain planarity	0.86 +- 0.11
Improper dihedral distribution	0.75 +- 0.03
Inside/Outside distribution	1.10 +- 0.02
