# Supplementary material for: DWNN, a novel ubiquitin-like domain, implicates RBBP6 in mRNA processing and ubiquitin-like pathways
Source: BMC Struct Biol. 2006 Jan 5;6:1. doi: 10.1186/1472-6807-6-1 (PMC1360078; doi:10.1186/1472-6807-6-1)
Supplement: Additional File 1 — Accession numbers of all sequences used in the multiple alignment in Figure 2. [file 1472-6807-6-1-S1.doc]

| Species | Accession Number |
| --- | --- |
| Homo sapiens | gi|20810414|gb|BC029352 |
| Mus musculus | gi|19484188|gb|BC025874 |
| Xenopus tropicalis | gi|38174410|gb|BC061346 |
| Fugu rubripes | emb|CAAB01001310 |
| Tetraoadon nigroviridis | emb|CAAE01011624 |
| Ciona intestinalis | gi|24615816|gb|BW201387 |
| Ciona savingnyi | gi|51718323|gb|BW538493 |
| Halocynthia roretzi | gi|6127995|gb|AV382938 |
| Danio rerio | TIGR:TC275204 |
| Anopheles gambiae | gi|31200960|ref|XM_309428 |
| Aedes aegypti | gi|46276209|gb|CB690872 |
| Bombyx mori | dbj|BAAB01116590 |
| Drosophila yakuba | gb|AAEU01005308 |
| Drosophila melanogaster | gi|4972739|gb|AF132177 |
| Drosophila psuedoobscura | gb|AAFS01000366 |
| Dictyostelium discoidium | gi|60469941|gb|EAL67923 |
| Oryza sativa | gi|32991777|dbj|AK106568 |
| Sorghum bicolor | gi|13238133|gb|BG356147 |
| Lycopersicon esculentum | gi|16245941|gb|BI931469 |
| Medicago truncatula | TIGR:TC98483 |
| Arabidopsis thaliana | gi|2245073|emb|Z97343 |
| Ceratopteros richardii | gi|9958581|gb|BE640922 |
| Kluyveromyces waltii | gb|AADM01000099 |
| Eremothecium gossypium | ref|NC_005783 |
| Saccharomyces kluyveri | gb|AACE01000494 |
| Saccharomyces castellii | gb|AACF01000070 |
| Saccharomyces mikata | gb|AABZ01000231 |
| Saccharomyces kudriavzevii | gb|AACI01000012 |
| Saccharomyces bayanus | gb|AACA01000247 |
| Saccharomyces paradoxus | gb|AABY01000117 |
| Candida glabrata | gi|50290108|ref|XM_447486 |
| Kluyveromyces lactis | gi|50305622|ref|XM_452772 |
| Debarymyces hansenii | ref|NC_006049 |
| Candida albicans | gb|AACQ01000005 |
| Schizosaccharomyces pombe | ref|NC_003423 |
| Gibberella zeae | gi|46136752|ref|XM_390068 |
| Magnaporthe grisea | gb|AACU01000995 |
| Coccidioides immitis | gb|AAEC01000163 |
| Coccidioides poadasi | gi|48512850|gb|CO005961 |
| Aspergillus nidulans | gi|49107796|ref|XM_411568 |
| Ustilago maydis | gi|49067291|ref|XM_397936 |
| Yarrowia lipolytica | gi|50543509|ref|XM_499921 |
| Cryptococcus neoformans | gi|41576458|gb|CF722299 |
| Encephalitozoon cuniliculi | GI|19173120|ref|NC_003238 |
| Vittaforma corneae | gi|17921328|gb|AZ694763 |
| Caenorhabditis briggseae | emb|CAAC01000060 |
| Caenorhabditis elegans | gi|15718172|emb|CAB04326 |
| Schistosoma mansoni | gi|34648112|gb|CD097622 |
| Chlamydomonas reinhardii | gi|6550584|dbj|AV396368 |
| Strongylocentrotus purpuratus | gi|56847034|gb|CX199610 |
